# Titanium Dioxide Nanoparticle: A Comprehensive Review on Synthesis, Applications and Toxicity

**DOI:** 10.3390/plants13212964

**Published:** 2024-10-23

**Authors:** Rakhi Chandoliya, Shivika Sharma, Vikas Sharma, Rohit Joshi, Iyyakkannu Sivanesan

**Affiliations:** 1 Department of Biotechnology, School of Bioengineering and Biosciences, Lovely Professional University, Phagwara 144411, India; chandoliyarakhi76@gmail.com; 2 Department of Molecular Biology and Genetic Engineering, School of Bioengineering and Biosciences, Lovely Professional University, Phagwara 144411, India; shivikasharma25@gmail.com (S.S.); biotech_vikas@rediffmail.com (V.S.); 3 Division of Biotechnology, CSIR-Institute of Himalayan Bioresource Technology, Palampur 176061, India; rohitjoshi@ihbt.res.in; 4 Department of Environmental Health Science, Institute of Natural Science and Agriculture, Konkuk University, Seoul 05029, Republic of Korea

**Keywords:** titanium dioxide, nanoparticle, growth-promoting factors, toxicity

## Abstract

Nanotechnology has garnered significant interest worldwide due to its wide-ranging applications across various industries. Titanium dioxide nanoparticles are one type of nanoparticle that is commonly utilised in everyday use and can be synthesized by different techniques using physical, chemical and biological extracts. Green synthesis is an economical, environmentally benign and non-toxic method of synthesising nanoparticles. Titanium dioxide nanoparticles have a positive impact on plant physiology, particularly in response to biotic and abiotic stresses, depending on various factors like size, concentration, exposure of the nanoparticles and other variables. Further, titanium dioxide nanoparticles have many applications, such as being used as nano-fertilizers, adsorption of heavy metal from industrial wastewater and antimicrobial activity, as discussed in this review paper. Previous studies investigated whether titanium dioxide nanoparticles also induce genotoxicity may be due to mishandling procedure, exposure time, size, concentration and other variables. This is still contradictory and requires more research. The present review is a pragmatic approach to summarize the synthesis, application, nanotoxicity, genotoxicity and eco-friendly method of nanoparticle synthesis and disposable.

## 1. Introduction

Among the most significant areas of contemporary research is nanotechnology, which is concerned with matter manipulation at the nanoscale. It is a multidisciplinary discipline with multiple applications in industries like agriculture, energy, textiles, medicine and autos [1]. Nanoparticles are defined as particles with a size between 1 and 100 nm; however, depending on their possible application, particles larger than 100 nm may potentially fall into this material class. Numerous metallic nanomaterials, such as those composed of copper (Cu), titanium dioxide, silver (Ag), zinc (Zn), iron (Fe) and others, appear to have both helpful and detrimental effects on plants, according to recent studies; nevertheless, more thorough investigation is needed to obtain more information [2]. As the main producers in food webs, plants provide important pathways for the bioaccumulation of engineered nanomaterials (ENMs). When ENMs are used excessively or inappropriately, they can have negative impacts on the air, water and soil, which can lead to the creation of main environmental reservoirs [3]. In recent years, titanium dioxide nanoparticles have become one of the most commonly used nanomaterials in commerce [4]. In 2014, the United States Geological Survey (2015) estimated that 1.31 million tonnes of titanium dioxide pigment were produced domestically worldwide. Roughly 10,000 tonnes of titanium dioxide nanoparticles are utilised annually in the paint, coatings, solar cell, cosmetic and cement industries [4], whereas an estimated 1.1 million tonnes of titanium dioxide pigment were produced domestically in 2021. The US was a net exporter of titanium dioxide pigments despite being mostly dependent on imports of titanium mineral concentrates. Titanium pigment exports rebounded greatly in 2021, following a multiyear low in 2020. It was predicted that 3.7 million tonnes of titanium dioxide pigment will be produced in China (U.S. Geological Survey, Mineral Commodity Summaries, January 2022). Because of its strong oxidation properties, high refractive index, low cost, remarkable chemical stability and oxygen vacancies in its lattice, titanium oxide nanoparticles are produced annually and are one the most versatile and prominent oxides. One of the most important characteristics of titanium dioxide nanoparticles for their usage as semiconductors in optical industrial applications is their broadband gap. Titanium dioxide nanoparticles have special electrical and/or ionic properties that allow for further customization for use in sensors and electronic devices. They can be utilized as a pigment in the paint industry and are a white, water-insoluble powder with a very high refractive index (*n* = 2.4). Three polymorphic forms of titanium dioxide are found in nature: rutile, anatase and brookite. These forms have crystalline structures and are widely employed in the gemstone industry. Titanium dioxide nanoparticles also have a wide range of practical uses in the food additive industry, cosmetics, anti-bacterial and anti-microbial agents, photocatalysts to break down wastewater pollutants and sensors [5]. Everyday use involves the functionalization of numerous items with titanium dioxide nanoparticles. Titanium dioxide nanoparticles are found in household items like toothpaste, shaving creams, sun protection creams, shampoos and conditioners [6]. Titanium dioxide nanoparticles are also frequently employed as food additives to improve the flavor, color and brightness of a range of food items [7]. Titanium dioxide nanoparticles are widely used in industry, and as a result, they are discharged into the air, water and soil. As a result, it is urgent to ascertain any potential effects on individuals and the biosphere. Numerous investigations have demonstrated that nanoparticles have harmful impacts on higher plants, invertebrates and microbes. Numerous types of plants have been studied in relation to titanium dioxide nanoparticle interactions; depending on a variety of circumstances, the effects may be beneficial or detrimental [8]. Titanium dioxide nanoparticles produced elevated amounts of reactive oxygen species, which rendered the marine microalga *Nitzschia closterium* hazardous [9]. In maize, too, harmful impacts from titanium dioxide nanoparticles have been documented. Nonetheless, titanium dioxide nanoparticles have produced positive effects on a variety of plant species, including *Vicia faba*, *Spinacia oleracea* and *Vigna radiata* [10,11,12]. The potential of titanium oxide nanoparticles in the food and agriculture sectors has made them more well-known. The primary functions of most researched nanoparticles include scavenging reactive oxidative radicals and activating antioxidant enzymes. Engineered nano-titanium dioxide primarily generates nanoparticles with a variety of uses in the food and agricultural sectors. Titanium dioxide nanoparticles have been demonstrated in several recent research to improve the development and yield characteristics of several stressed plants. Two main methods that titanium dioxide nanoparticles use to achieve their advantageous effects are the scavenging of reactive oxidative species and the activation of antioxidant enzymes [13]. A collection of current resources about titanium dioxide nanoparticles and their interactions with plants has been developed, with consideration to the sometimes-contradictory roles that these particles play. The purpose of this study is to discuss the synthesis of titanium dioxide nanoparticles and their effect on plant growth and development and how titanium dioxide nanoparticles recycled and disposed without harming the environment.

## 2. Synthesis of Titanium Dioxide

**Figure 1:**
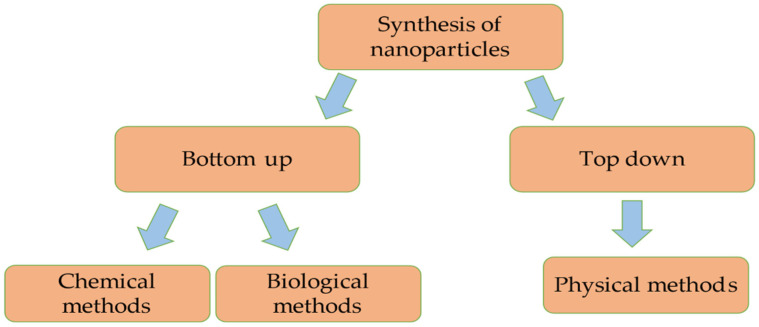
Classification of synthesis of nanoparticles.

Conventional techniques for synthesizing metal oxides can be classified into two categories: top-down and bottom-up. In the top-down method, a variety of physical techniques, including etching, sputtering, pulse laser ablation, evaporation-condensation and milling, are used to break down bulk macroscopic particles into nanoscopic particles [14,15]. On the other hand, in a bottom-up method, many techniques and procedures such as chemical vapour deposition, sol-gel process, flame spraying, sonochemical, spinning, hydrothermal, green synthesis, etc., are used to generate nanosized particles after the atomic nuclei are brought together by a self-assembly process. The bottom-up technique has garnered attention because it offers size and structural control over nanoparticle synthesis. Although chemical processes are less expensive, they have limitations in terms of mass manufacturing, high energy, temperature and pressure requirements, as well as the use of volatile and poisonous chemicals that endanger human health and the environment. Therefore, scientists have investigated a novel biological way of synthesising nanoparticles, which is non-toxic, safe for the environment, takes less energy for cost-effective production and employs less expensive chemicals (Figure 1).

### 2.1. Chemical Synthesis of Titanium Dioxide Nanoparticles

**Figure 2:**
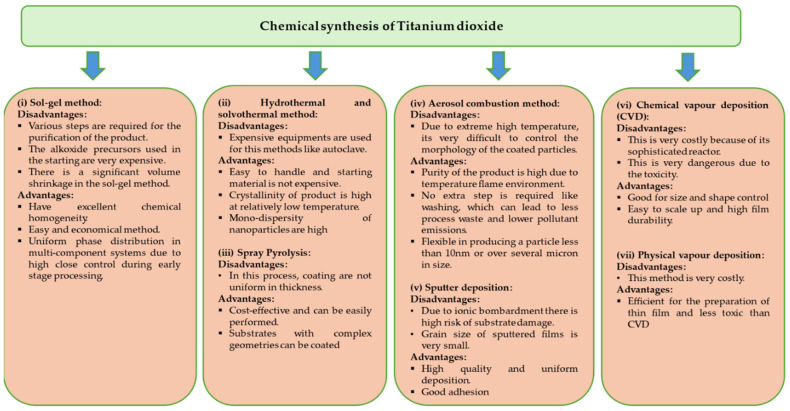
Various methods of chemical synthesis of titanium dioxide nanoparticles and their disadvantages.

Titanium dioxide nanoparticles are synthesised using a variety of chemical synthesis methods, including the co-precipitation method [16], sol-gel process [17], solvo-thermal method [18] and hydrothermal method [19]. Although the chemical method of creating titanium dioxide nanoparticles is popular because it is simple to use and allows for control over the size and form of the nanoparticles, there are drawbacks, including high energy costs, high temperature and pressure, ecotoxicity and environmental sustainability. This also restricts their ability to be produced in large quantities and used in a variety of industries [20]. Numerous investigations on chemically synthesized titanium dioxide nanoparticles have been conducted, and their drawbacks and limits have also been discussed, as shown in Figure 2. The synthesis of nanoparticles using both hydrothermal and solvo-thermal techniques requires the use of an autoclave that operates at high pressure and temperature. However, the solvo-thermal process uses non-aqueous solvents, and the hydrothermal method uses aqueous solvents. According to Nasirian and Mehrvar (2018), the hydrothermal approach to producing titanium dioxide nanoparticles is expensive since it requires a lot of energy and higher temperatures and pressures. Furthermore, it is a labor-intensive procedure that produces fewer titanium dioxide nanoparticles [21]. Like this, Solvo-thermal systems operate at high temperatures and pressure (above the water’s boiling point; pressure < 1 atm). Additionally, the procedure takes a lot of time and requires expensive equipment, such as the autoclave’s high maintenance costs [22]. Additionally, the process becomes more difficult due to the use of surfactants, which also introduce impurities [23]. Because it employs organic solvents, solvo-thermal is seen to be the least hazardous method in terms of environmental toxicity, whereas hydrothermal uses surfactants, which have been shown to be harmful to ecosystems, particularly aquatic life. Titanium dioxide nanoparticles are created by various deposition procedures, which include electrophoretic, thermal plasma and spray pyrolysis methods for synthesis. This spray pyrolysis method is frequently used to synthesise powders and dense, uniform films, but it has drawbacks in terms of low-cost effectiveness because it necessitates sustaining low temperatures, which is an expensive and energy-intensive procedure. Furthermore, it has been determined that this limits the ability to control the characteristics of powders and prevents the manufacturing of titanium dioxide nanoparticles using spray pyrolysis at a large scale [24]. Environmental issues relating to particulate matter emissions require costly equipment for control. Similarly, it is known that the electrophoretic synthesis of titanium dioxide is simple and saves time, but it is rigid in its choice of solvent—water [25]. Furthermore, these non-aqueous solvents’ evaporation pollutes the air and endangers people’s health [26]. Like this, it has been reported that the microwave-assisted method for titanium dioxide synthesis is a widely accepted technique. This is because the reaction mixture is heated uniformly and quickly to the required temperature, saving time. However, the microwave-assisted methods are not cost-effective because they require high-power microwave heating, which is also energy intensive. Furthermore, the growth of titanium dioxide particles in relation to time cannot be monitored using this method. Furthermore, mass manufacturing of titanium dioxide particles is not possible with microwave-assisted synthesis [27]. To create highly crystalline nanoparticles, the sol-gel production process for titanium dioxide has been extensively studied. It entails sol formation, gelation and solvent removal [17]. However, the sol-gel procedure is notoriously costly because of the high cost of raw materials, volume loss and cracking of the titanium dioxide produced during the drying process used to remove organic material [28].

### 2.2. Biological Synthesis of Titanium Dioxide Nanoparticles

The production of titanium dioxide nanoparticles using chemical methods has been found to be environmentally hazardous due to the high temperature and pressure involved, which also limits the amount of titanium dioxide that can be produced in large quantities. Since green nanotechnology uses reducing agents sourced from biological sources and may be used to synthesis numerous metallic compounds, it has been investigated as an eco-friendly and alternative method for the synthesis of titanium dioxide nanoparticles [29]. Additionally, less harmful and costly chemicals are employed in the synthesis because plants, their fruit extracts, waste products and microorganisms are used [30]. Additionally, it can be used to produce titanium dioxide nanoparticles on a large scale at a reasonable cost [20]. To create a stable, appropriately sized and easily dispersed nanoparticles with little energy use, green synthesis techniques are essential [31]. Using the green approach, titanium dioxide nanoparticles were synthesised and examined using Fourier transform infrared (FTIR), transmission electron microscopy (TEM), selected area electron diffraction (SAED), energy-dispersive X-ray spectroscopy (EDS or EDX), X-ray diffraction (XRD), ultraviolet-visible (UV-vis) and scanning electron microscopy (SEM) [29].

#### 2.2.1. Synthesis from Green Plants Extract Nanoparticles

Plant extract is typically the most important ingredient in the synthesis of titanium dioxide nanoparticles due to its safety and feasibility benefits. Different shaped and sized nanoparticles are derived from plants and their diverse sections, including the stem, leaves, latex, flowers, seeds and roots. Most studies have been done on plant components like leaves, seeds and flowers. The primary reason they are favoured is due to their great ability to lower metal ions [32]. Plant accessibility and handling safety are further benefits. Polysaccharides, Alkaloids, diterpenoids, salicylic acids, lactones, glycosides, amino acids, steroids, etc., are examples of bioactive substances derived from plant sources that function as reducing and stabilising agents. Green chemistry has been effectively used to synthesise nanoparticles such as aluminium, iron, zinc, manganese, copper, titanium and cobalt. Green synthesis is becoming more and more popular over chemical approaches due to its many benefits, including affordability, reduced toxicity, environmental friendliness and improved uses. Green synthesis can be divided into three primary groups: (a) Making use of microorganisms such as bacteria, yeast and fungus; (b) making use of plant components and the extracts of plants; and (c) making use of templates such as viruses and deoxyribonucleic acid (DNA). Plants and their extracts have become more and more popular over time. Numerous uses resulting from the effective biosynthesis of titanium dioxide nanoparticles are investigated. Because leaves are a rich source of metabolites, they are used for extracts more frequently. It is more practical to extract them without creating any harmful substances [14]. For example, Nabi et al. (2019) observed titanium dioxide nanoparticles made with a cinnamon powder extract utilising the green synthesis approach. It was determined that the synthesis procedure was simple, workable and economical. Additionally, it was discovered that the synthesised nanoparticles were the anatase phase of titanium dioxide, including spherical particles with O_2_ vacancies and self-trapped excitons for photocatalytic activity in applications related to solar cells, in addition to being band gap functional under visible light [33]. In a further investigation, *Syzygium cumini* leaf extract was utilised to create spherical and irregularly aggregated titanium dioxide particles [34]. It was found that the process was affordable, non-toxic, simple to use and good for the environment. It was used with 82.53% efficiency to remove lead from wastewater [34]. Similarly, extract from *Moringa oleifera* was used to synthesise tetragonal titanium dioxide nanoparticles, which was found to be a simple, inexpensive, eco-friendly and time-saving synthesis technique [14]. The biological method was used by [35] to synthesise titanium dioxide nanoparticles from orange peel extract. Compared to chemically generated nanoparticles, it was reported to be a more environmentally friendly method with better outcomes in antibacterial, humidity and cytotoxicity sensors. Similarly, Abisharani et al. [36] reported on the biosynthesis of tetragonal titanium dioxide nanoparticles using *Cucurbita pepo* seed extract. Titanium dioxide nanoparticles were synthesised and used to cleanse water sources without causing environmental harm [37]. It has been observed that the synthesis of titanium dioxide from *Trigonella foenum-graecum* leaf extract is faster and cleaner. Furthermore, it was found that titanium dioxide nanoparticles produced using an affordable and environmentally acceptable method might be used to produce titanium dioxide nanoparticles on a big scale [31]. Moreover, the application of *Cynodon dactylon* leaf extracts demonstrated the benefits of ease of use, economy and suitability for medical usage. The synthesised titanium dioxide nanoparticles had good light absorption and refractive index, were non-toxic and were less expensive [38]. Moreover, the authors described the biological techniques for mass-producing titanium dioxide nanoparticles as *Trigonella foenum-graecum* [31]. Rod-shaped titanium dioxide nanoparticles synthesised using aloe extract for photocatalytic applications. It was determined that the synthesis technique was simple, environmentally benign and highly active. Pomegranate peel was also employed in the manufacture of titanium dioxide nanoparticles, which were used to purify water sources without harming the environment. For the first time, a green and environmentally friendly process was utilised to biosynthesize titanium dioxide quantum dots (TiO_2_Qds) from watermelon peel waste. Full characterization of the biosynthesized TiO_2_Qds was performed with UV-visible, FTIR, XRD, SEM, EDX, mapping, TEM and thermal gravimetric analysis (TGA). The synthesised TiO_2_Qds have an average particle size of 7 nm and a polycrystalline crystal structure, according to the characterisation of the material. Watermelon peel waste is used to biosynthesize TiO_2_Qds, which exhibit strong biocompatibility and antibacterial, antioxidant and anticancer properties [39]. Various sizes of titanium dioxide nanoparticles are synthesized from different plant species, as shown in Table 1.

#### 2.2.2. Synthesis from Microbial Extract

Microbes are significant nanofactories with enormous promise as economical and environmentally benign instruments that eliminate the need for hazardous chemicals and the high energy required for physicochemical synthesis. Both intracellular and extracellular manufacturing of metal oxide nanoparticles has been accomplished using a variety of microorganisms, including bacteria, fungi and yeast. Transporting the metal ions into the microbial cell and creating the nanoparticles while the enzyme, coenzymes and other biomolecules are present within the cell are the two steps of intracellular synthesis. Metal ions are trapped on the surface of the microbial cell during extracellular production.

In addition to lowering the metal ions, the surface-available proteins and enzymes stabilise the nanoparticles. Extracellular synthesis, on the other hand, has several advantages over the intracellular pathway, including the ability to produce huge amounts of nanoparticles and the elimination of several synthesis stages needed for nanoparticle recovery [49]. However, this approach has a number of shortcomings: (a) The microorganisms must be screened; (b) the culture broth and the entire process must be closely monitored; and (c) it is challenging to control the size and morphology of the nanoparticles. Because of their inherent metabolic processes and enzyme activity, not all bacteria are able to synthesise nanoparticles. Because of this, carefully selecting the right bacteria is essential to producing nanoparticles with precise size and morphology [49]. Titanium dioxide nanoparticles have been discovered to exist in a range of sizes and forms due to the fact that fungi include metabolites and enzymes that allow them to break down bulk salts into particular ions [20]. Baker’s yeast was used as a low-cost way to synthesise anatase tiny-sized titanium dioxide nanoparticles with excellent purity and stability when using bacteria to produce titanium dioxide. The effective antibacterial activity of baker’s yeast was discovered [50]. In a different research, clean, eco-friendly, less poisonous and costlier titanium dioxide nanoparticles were created using the *Streptomyces* sp. bacterium. It was noted that the procedure for antimicrobial antibiofilm activity was quick and easy [51]. To improve photocatalytic dye treatment with 78% efficiency in a short length of time using only 0.3 g titanium dioxide, a cost-effective biosynthesis approach was investigated for *Acinetobacter baumannii*, *Bacillus subtilis*, *Aeromonas hydrophila* and *Lactobacillus* sp. bacteria were among those whose mass production of titanium dioxide was shown to be simple, easy, repeatable and economical to synthesise. *Planomicrobium* sp. biomass was shown to be useful for the environmentally benign and less expensive synthesis of titanium dioxide nanoparticles in a different investigation on green synthesis. Additionally, the protein from the employed bacterium added to the stability of titanium dioxide nanoparticles.

#### 2.2.3. Synthesis from Other Biological Sources

**Figure 3:**
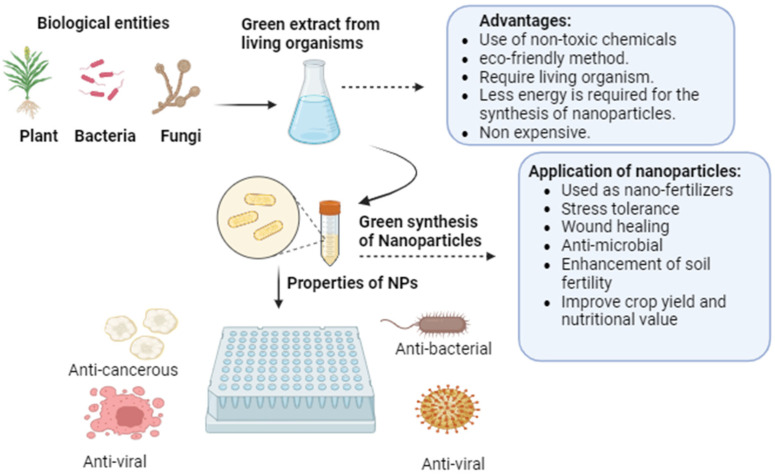
Biological synthesis of titanium dioxide and its properties.

Titanium dioxide nanoparticles are synthesised using a variety of biological sources besides plants and microbes. Although they have not been well studied, biological derivations for synthesis have been found to have advantages in terms of nanoparticle production that are both environmentally friendly and cost-effective. For example, it has been reported that starch may be used to easily synthesise green titanium dioxide with good photocatalytic capabilities and environmental friendliness [52]. In a similar manner, consistent titanium dioxide nanowire efficiency was synthesised using cellulose fibers. It also demonstrated the benefits of recovering cellulose fibers without altering their shape. Additionally, as a benefit of green synthesis, biological materials like eggshells were utilized for a straightforward and biomimetic approach to create an organized tube network of titanium dioxide. Moreover, the particles’ high surface area and porosity made them useful for a variety of applications using green synthetic titanium dioxide. Additionally, using egg albumen was thought to be a reproducible, affordable and environmentally friendly way to synthesise titanium dioxide. In a different investigation, egg albumen served as a dependable and affordable gelling agent for the synthesis of titanium dioxide [53]. Titanium dioxide was synthesised utilising two polymers made of amino acids, following a bioinspired approach. It was discovered to be a gentle method that changed the titanium dioxide’s anatase phase into rutile, which was advantageous for its application [54]. Titanium dioxide nanoparticles were created by utilising the inexpensive, easy and straightforward starch synthesis method. It was discovered that the synthesised rutile phase could be used for UV light protection. In a similar manner, titanium dioxide particles were synthesised using lysozyme. Compared to titanium dioxide particles that are manufactured normally, Lyzome is a simple method that has effective photocatalytic capacity and has assisted in reducing agglomeration [55]. Rice straw has been used for titanium dioxide synthesis due to its advantages as a soft, simple and environmentally friendly template. Similarly, gelatin was thought to be a biological source for the easy and straightforward production of titanium dioxide nanoparticles, which had a higher surface area and better capacity to store hydrogen thanks to the usage of gelatin, as well as in the application of peptides [56]. According to reports, arginine and protamine can be used in the environmentally friendly synthesis of titanium dioxide thanks to their affordability and ease of use (Figure 3).

## 3. Applications of Titanium Dioxide

### 3.1. Environmental Stresses and Effect of Titanium Dioxide Nanoparticles on Crop Production

**Figure 4:**
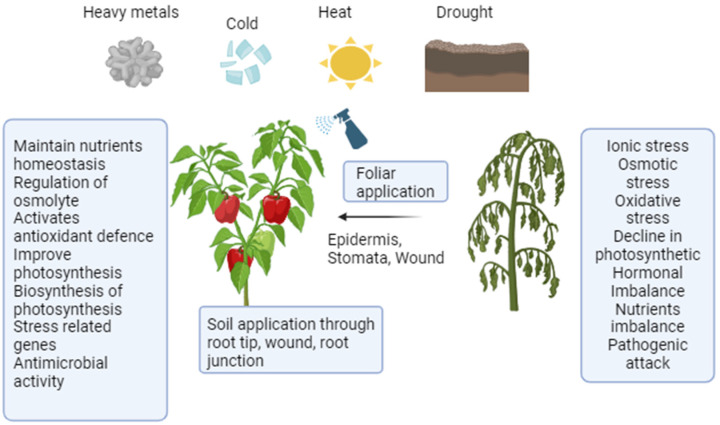
Application of Titanium dioxide nanoparticles.

In agriculture, the main determinants of crop yield and fruit quality are the environmental conditions. Numerous abiotic challenges, including heat, drought, nutrition and salt stresses, were brought on by these environmental changes. Crop damage caused by environmental pressures is far more severe than crop damage caused by physiochemical, mechanical and biochemical causes combined. In response to these types of adverse environmental conditions, plants initiate a range of physiological responses and develop the ability to biosynthesize distinct phenolics, which fully release increased antioxidant capacity and ultimately enhance plant performance in difficult environments [57]. There are various conflicting reports about the effects of titanium dioxide nanoparticle treatment on plant performance. Lipid peroxidation and the ensuing decrease in development have also been seen in *Nitzschia closterium* treated with titanium dioxide nanoparticles [58,59]. Furthermore, it was revealed that titanium dioxide nanoparticles inhibited the growth and decreased the phenolic compound contents of two microbe species while increasing the phenolic compound contents of other species. An increase in the number of phenolic compounds, antioxidant capacity and essential oil content in cotton, dragonhead and *Vitex* plants treated with titanium dioxide nanoparticles [60]. There have also been reports of titanium dioxide nanoparticle exposure having mitigating effects on chickpea membrane damage [61,62,63]. Titanium dioxide nanoparticles have recently demonstrated a successful rise in thymoquinone concentrations in black cumin by enhancing the expression of the relevant metabolic gene pathways [64]. The concentration and kind of nanoparticles, as well as abiotic and biotic parameters that have been evaluated, could all be responsible for these inconsistent outcomes. Strong irradiance exposure to titanium dioxide nanoparticles caused a multitude of morphological and physiological effects in tomato (*Solanum lycopersicum* L.) plants, including increased fruit and blossom output, enhanced carotenoids as well as anthocyanin and also increased activity of enzymes. The most notable finding with nano titanium dioxide was the high production of fruit, which was caused by stress-induced reaction accelerating propagation [65]. In addition to the impacts of external stressors on agricultural yields, heavy metals present unfavourable circumstances for soil and plants alike (Figure 4).

The need for nanoparticles to reduce the amount of metals that plants take up when they are planted in contaminated soil that contains heavy metals is growing. Numerous investigations have shown that metal oxide nanoparticles may be a more effective way to prevent agricultural plants from absorbing metals [66,67]. Among the other known nanoparticles, titanium dioxide nanoparticles have been proven to be effective and can be used in agriculture, especially for the treatment of contaminated soils due to heavy metals. Previous investigations show that the nanoparticles have both positive and negative effects on plant development under different environmental circumstances [68,69]. Few studies, meanwhile, have shown how titanium dioxide nanoparticles affect plants’ ability to absorb heavy metals. Titanium dioxide nanoparticles impact crop physiological parameters that are dose-dependent, dependent on experiment type, size and targeted heavy metals [70]. Titanium dioxide nanoparticles accumulation of heavy metals in plants is dependent on the types of soil [71], which supports the application of these particles through hydroponic culture, foliar route seed priming and field experiments. In the end, it is beneficial to implement on a large scale where there is this kind of environmental issue because it has been demonstrated to be successful in lowering the targeted heavy metal through crops at a variety of concentrations and particle sizes. Seedling growth parameters of *Origanum majorana* L. were improved with the help of seed primming with a concentration of 20 mg/L and 40 mg/L of the bulk and titanium dioxide nanoparticles by improving the scavenging activity of free radicles in salinity stress [72]. By upregulating ABC transporters and the synthesis of phenylpropanoid pathway products such flavones, isoflavones, flavonols and anthocyanins, which contribute to plant defense against environmental stressors, titanium dioxide nanoparticles also improve intracellular sequestration [73]. Titanium dioxide nanoparticles improved the production of proline, an osmatic regulator that helps reduce the toxicity of cadmium (Cd) in plants. Therefore, titanium dioxide nanoparticles may reduce the negative effects of Cd stress and increase coriander yield [74]. Osmotic stress caused by polyethylene glycol (PEG) and Nickel was countered by the increase in proline and carbohydrate levels, which helped the seedlings maintain their optimal level of hydration. Titanium dioxide enhanced the biosynthesis of H_2_S and K+ retention via regulating cysteine biosynthesis and H+-ATPase activity. In brief, titanium dioxide maintains redox homeostasis and the normal functioning of nitrogen and carbohydrate metabolism, which results in the protection of cucumbers. The intake of the stressed seedlings with titanium dioxide improved the accumulation of phytochelatins and the activity of the glyoxalase system enzymes that provided further defense against the metal and toxic methylglyoxal [75]. In another study, it was found that titanium dioxide-treated tomato plants have increased relative water content and decreased proline and malondialdehyde content under drought conditions [76]. Titanium dioxide nanoparticles have great potential under environmental stress, such as reduced arsenic toxicity in *Vigna radiata* [77], as shown in Table 2 and Table 3.

According to Omar et al., *Vicia faba* was treated with different concentrations (10 or 20 nm) of titanium dioxide nanoparticles to overcome salinity stress by upregulated heat shock protein, which reduces the osmotic and toxic effects of salinity stress in plants. Salinity stress is responsible for high chromosomal aberrations and DNA damage, decreased dry weight and increased electron leakage [78]. Titanium dioxide nanoparticles increased the rosmarinic acid content in *Dracocephalum kotschyi* transformed roots by upregulating the phenylalanine ammonia-lyase (pal) and rosmarinic acid synthase (ras) genes. Furthermore, titanium dioxide nanoparticles are also responsible for the production of anti-cancerous flavonoids like cirsimaritin and xanthomicrol [79]. Similarly, by upregulating the activity of adenylytransferase (*APT*), adenosine-5′-phosphosulfate reductase (*APR*) and sulfite reductase (*SiR*) enzyme, titanium dioxide nanoparticles are responsible for the alleviation of tetracycline toxicity in *Arabidopsis thaliana* [80].

### 3.2. Impact of Titanium Dioxide Nanoparticles on Sorption of Heavy Metals from Wastewater

One of the main causes of water pollution in the world is thought to be due to heavy metals in sewer water. The main sources of water contamination, particularly heavy metals, are home sewage sludge and industrial effluents. Concerns about water pollution are growing quickly, and they are having an impact not only on human health but also on the global economy and sustainable development. They affect both the local living biota and human health because of their nonbiodegradable impacts. Because heavy metals bind to the same places as essential metal ions do, they induce the destabilization of structures and biomolecules, which in turn contributes to mutagenesis, tumours and genetic diseases [59]. The heavy metal ions found in wastewater that are particularly concerning are arsenic, chromium, cadmium, mercury and lead since they are highly harmful to living things. These heavy metals can enter the body through food, drink and the air. Even minute concentrations of these substances can have harmful consequences that can last for a long time [101,102]. Furthermore, heavy metals have an impact on crop quality, yield and growth. The development of efficient techniques for wastewater treatment takes into account a number of techno-economic, environmental and social factors. As heavy metals are difficult to remove by biological, physical, or chemical processes, removal of these contaminants requires both immersion and isolation [66]. Plant-based degradation, conventional approaches, microbiological treatments, wastewater treatment and heavy metal remediation using nanomaterials are some of the remediation techniques. Several researchers have reported on a variety of metallic and metal oxides [103].

In treatment methods, heavy metals from residential or industrial wastewater can be sorbent thanks to the unique and appealing features of nanoparticles. Nanomaterials excel in separation technology due to their small size, high specific surface area and distinctive morphological features. Furthermore, the large surface area and increased surface-to-volume ratio of the nanoparticles create more sites on the sorbent’s surface, boosting its sorption capacity [104]. A variety of nano-sized materials have been used as adsorbents to remove heavy metal ions from water or wastewater, including metal oxide, carbon-based nanoparticles, nano-clays and nanocomposites. Titanium dioxide nanoparticles have attracted a lot of attention because of their remarkable chemical and physical properties, which include their strong reducing and oxidizing capability, high permeability and distinctive optical and electrical properties. It is an excellent semiconductor with a wide range of uses that is mostly known in light research. In addition, titanium dioxide nanoparticles find extensive use in the food sector, personal hygiene products, wastewater treatment for pathogen inactivation and a wide range of building materials, including paints, plasters and tiles [105]. Hence, titanium dioxide nanoparticles are a material that is naturally safe and has very little photocatalytic activity in visible light and a strong one in UV light [106]. Titanium dioxide nanoparticles have the potential to alter the toxicity and bioavailability of pollutants, such as Cadmium, through interactions [107]. The nanosheet made from titanium dioxide has the potential to absorb photo light and restore its infusion flux in situ when applied under visible light treatment.

### 3.3. Photolytic Properties of Titanium Dioxide Nanoparticles

Numerous industrial and residential wastewater systems may contain hazardous colours and nitroarene chemicals, among other toxic water contaminants. They will be able to stay in the environment for a long time and threaten aquatic life in various ways due to their poor solubility and great persistence. As of right now, metallic nanoparticles’ ‘s unique shapes and potent catalytic capabilities have been proven. Because of their large surface area, some metal nanoparticles can offer the best heterogeneous catalytic activity. Many scientists have documented the high-quality photocatalytic activity of green synthesized nanoparticles made from titanium dioxide to destroy various colours and other organic compounds in wastewater [52]. The greatest aquatic pollutant that causes eutrophication in different water bodies, phosphate, was successfully removed utilising phytochemical-mediated titanium dioxide nanoparticles mediated by *Prunus yedoensis* [108]. Functional groups included in leaf extracts have been identified using FTIR spectroscopy. The produced titanium dioxide nanoparticles exhibited a spectrum with changes in the 1082 and 1377 cm^−1^ bands, a peak at 655 cm^−1^ and the stretching of the C–H bending mode of the remaining butyl group, which were attributed to the bonding of metals. As evidenced by the 1626 cm^−1^ peak, the absorbed water molecules’ O-H stretching vibration increased the photocatalytic activity effectively [108]. In a more recent development, the produced titanium dioxide nanoparticles exhibited a spectrum with changes in the 1082 and 1377 cm^−1^ bands. A peak at 655 cm^−1^ was synthesised using *Jatropha curcas* leaf extract and utilised in the photocatalytic degradation of effluent from tanneries. Additionally, FT-IR spectroscopy was utilised to determine the potential biomolecules through the examination of chemical groups present in a *Jatropha curcas* leaf extract that is responsible for the reduction or capping of metallic ions (Ti^+4^) precursor, which is essential for the synthesis of nanoparticles [109]. Moreover, a wide band at 3495 cm^−1^ indicated the presence of hydroxyl groups, which may have resulted from the leaf extract’s phenol content. The polyphenolic tannins in the leaf extract, which might cap the surface of the green synthetic titanium dioxide nanoparticles, are what probably caused the phenolic group to emerge. This study successfully constructed a self-designed solar-photo catalytic parabolic trough reactor; chromium removal was reported to be 76–82%, and considerable chemical oxygen demand (COD) reduction was also noted [109]. Their results indicate that titanium dioxide nanoparticles have extraordinary activity in the realm of photocatalysis. Recalcitrant organic and inorganic pollutants can be effectively removed, degraded and detoxified from industrial wastewater using a photocatalytic treatment method that uses nanosized titanium dioxide_._ Titanium dioxide is known for its valence band and conduction band, two distinct band types. Additionally, its band gaps for the anatase, brookite and rutile phases are 3.0, 3.4 and 3.3 eV, respectively. When a specific wavelength of radiation is applied to titanium dioxide, it can cause an electrical excitation that results in the creation of electron-hole pairs. These pairs, when combined with oxygen and water, form oxidative species like OH, O^2−^, H_2_O_2_ and O_2_, that can efficiently degrade organic contaminants. Titanium dioxide’s very low band gap energy allows it to easily produce OH species when exposed to UV or visible light [110]. Excitation of titanium dioxide electrons from the valence band to the conduction band starts the process, which is followed by several further reaction steps as the light energy overcomes the band gap energy. These investigations showed that green-synthesized titanium dioxide nanoparticles had a greater tendency to absorb light than chemically synthesized ones because of the addition of phytochemicals in the crystal lattice. Considering these elements, it is also beneficial to correlate the catalytic behaviors of titanium dioxide nanoparticles synthesised utilizing plant extracts with their physical and chemical properties to clarify the photocatalytic activity of these nanoparticles. These encouraging results may support future studies that show these compounds’ catalytic properties in the breakdown of other pollutant classes, including amines, polyaromatic hydrocarbons, pesticides and phenols.

### 3.4. Antimicrobial Activity of Titanium Dioxide Nanoparticles

Plants and microorganisms, such as bacteria, fungi and algae, which function as effective reducing and capping agents to produce nanoparticles, are used to manufacture biosynthesized nanoparticles. Numerous metal nanoparticles’ effects on distinct bacterial strains have been documented in the literature. Similarly, because of their strong oxidising potential, titanium dioxide nanoparticles might exhibit biocidal properties that are beneficial to the environment. Every year, dangerous bacteria such as *Clostridium difficile*, *Staphylococcus aureus*, *Escherichia coli*, *Burkholderia cepacia*, *Pseudomonasa aeruginosa* and *Klebsiella pneumoniae* cause microbial infections that can cause severe diseases in human beings. The main tools for solving this issue are antimicrobials, antifungals and antibiotics. However, several bacterial strains are now far more resistant to these drugs, which is why researchers are currently focusing on finding new antimicrobial compounds. The antibacterial characteristics of metal oxide nanoparticles have been extensively studied due to their advantageous effects, yielding positive findings. Titanium dioxide nanoparticles are among the antibacterial nanoparticles that have been the subject of extensive research recently in this regard. ROS are created when titanium dioxide nanoparticles encounter microbial cells [6]. Furthermore, phospholipid oxidation lowers adhesion and disturbs the ion balance, and these ROS can effectively eliminate bacteria by impairing the cohesion of their cell walls. It also modifies the morphologies of macromolecules and suppresses the cytosol’s respiratory cytosolic enzymes, which significantly impacts cellular integrity and gene expression. Furthermore, it lessens cellular contact amongst cells and the use of phosphate [6]. Both chemically and biologically manufactured titanium dioxide nanoparticles have the same ability to eradicate microorganisms. Titanium dioxide nanoparticles were created using *Psidium guajava* plant extract. The synthesized titanium dioxide nanoparticle’s XRD pattern demonstrated the presence of both rutile and anatase forms. The primary functional groups of the nanoparticles were identified by the FTIR peaks of the leaf extract. The alcohols (free -OH) peak is located at 3420 cm^−1^, the intramolecular bonded (weak) peak is at 3410 cm^−1^, the strong intramolecular bonded peak is at 3425 cm^−1^, the alkenes peak is at 2922 cm^−1^, the carboxylic acid peak is at 2917 cm^−1^, and the nitro compound (symmetrical stretch) peak is at 1659 cm^−1^. The bioreduction of TiO (OH)_2_ to titanium dioxide nanoparticles produced these functional groups. With an average size of 32.58 nm, the plant-synthesised titanium dioxide nanoparticles were highly polydisperse in terms of particle size. The produced titanium dioxide nanoparticles showed the highest inhibitory zone against *Staphylococcus aureus* (25 mm) and *Escherichia coli* (23 mm) [111]. Consequently, nanoparticles produced biologically typically have far more antimicrobial activity. In a process that is still being investigated, the remarkable antibacterial activity of plant extracts is thought to be due to the capping agent they create. Because some extracts are used in the synthesis process to produce desired qualities, it is necessary to conduct a systematic investigation of the catalytic behaviour to determine how these properties relate. The plant extract-derived nanoparticles showed a comparatively smaller and more defined crystalline form (about 17.30 nm) than the chemically synthesised nanoparticles (particle size 21.61 nm) [112]. When the antibacterial activity was measured, the more environmentally friendly nanoparticles demonstrated more bactericidal activity than the chemically synthesised nanoparticles against both Gram-negative and Gram-positive bacteria. Mansoor et al. claim that titanium dioxide derived from *Bacillus subtilis* exhibits remarkable promise for the management of dental conditions [113]. Both procedures produced anatase crystalline structures. It was determined that the antibacterial activity of nanoparticles was significantly influenced by their structures, the biochemical composition of the microbial membrane and the bacterial shape. When compared to the conventional antibiotic disc, the titanium dioxide nanoparticles appeared to have greater antibacterial action [114].

## 4. Use of Titanium Dioxide as Nano-Fertilizers

The use of conventional fertilisers such nitrogen, phosphorous and potassium (NPK) fertilisers and mineral fertilisers is one of these techniques; nevertheless, reports of their efficacy indicate that it is approximately 25% and below 30%, respectively. Using materials with sizes ranging from 1 to 100 nm, nanotechnology is typically applied in agriculture to enhance the growth and functionality of plants and crops [115]. Because of their superior characteristics, such as increased surface area, reactivity and smaller particle sizes, engineered nanoparticles can be used as fungicides, germicides and nanofertilizers [116]. The advantages of nano-fertilizers over traditional fertilisers have been explored, including improvements in plant growth and nutrient availability. Nanoparticles that comprise fertiliser (also known as nanoscale fertiliser), conventional fertilisers coated with nanoparticles (also known as nanoscale coating) and conventional fertilisers with nanoscale additives (also known as nanoscale additives) are examples of nano-fertilizers created via nanotechnology. Research on nanofertilizers has shown that plant roots and leaves may absorb and transfer nanoparticles. After the nanoparticles are absorbed by the leaf cuticle, they go to the palisade and spongy mesophyll cells through the plant’s epidermal cell layer, where they are subsequently taken up by the vascular bundles. On the other hand, Nanoparticles pass via the xylem and phloem and enter the epidermal cell layer of plant roots after being absorbed by root hairs. Aside from this, symplastic and apoplastic channels enable their translocation to the cortex of roots [117]. The most common type of nanoparticles is bio-synthesized titanium dioxide, which is also known to be an efficient nanofertilizer with biochemical, physiological and morphological effects on the metabolic activity of plants due to increased biomass production [118,119]. Based on the stability, photoactivity, biocompatibility, structure and tunable hydrophilicity of titanium dioxide nanoparticles, using them to overcome concerns related to the environment and agriculture is a viable and sustainable alternative. Because porous absorbent nanoparticles that make up carbon and metallic nanoparticles that are unstable in water are less desirable, titanium dioxide nanoparticles make better nanofertilizers [120,121]. Furthermore, plants may withstand abiotic challenges by applying titanium dioxide nanoparticles, which start a defence mechanism in the plants. Additionally, plant species, the quantity of titanium dioxide nanoparticles applied, the size and form of titanium dioxide nanoparticles and environmental conditions all affect how tolerant a plant is to stressors. Titanium dioxide nanoparticles help boost photocatalysis because they increase plants’ ability to absorb sunlight, which encourages the light energy to be converted into chemical energy as well as active electrons. However, there have also been reports of time- and dose-dependent toxicity associated with titanium dioxide nanoparticles [121]. Titanium dioxide nanoparticles, for example, have been shown to promote red bean (*Vigna angularis* L.) growth. After one to three weeks of exposure to titanium dioxide nanoparticles, there were negligible adverse physiological effects and an improvement in the transport and uptake of nutrients. The study reported on the individual and cumulative exposure of red beans to Zinc oxide and titanium dioxide. The results indicated that a single exposure to titanium dioxide was more beneficial in mitigating oxidative stress, enhancing chlorophyll, promoting translocation and promoting root growth as well as photosynthesis [122,123,124]. Conversely, Zinc oxide was found to have minimal effects on improving root growth, even in conjunction with titanium dioxide [125]. Similarly, in a different investigation, the *Aspergillus flavus* TFR 7 fungus was used to biosynthesize mung beans via extracellular enzyme secretions. Likewise, the length of shoots and roots, as well as the quality of wheat grains, were enhanced by titanium dioxide nanoparticles (50 mg/kg). Furthermore, absorption and bioavailability of micronutrients like copper, aluminium, iron and zinc were enhanced, as was phosphorous (P) absorption, even in the absence of P-containing fertiliser [126]. Titanium dioxide nanoparticles applied topically at a concentration of 10 ppm have been shown to promote shoot development and the quantity of essential oils under typical circumstances. Furthermore, it was observed that applying titanium dioxide minimised membrane damage and reduced oxidative stress in situations where there was insufficient water [127]. Similarly, under cold stress, or 4 °C, licorice was investigated for the physiological and biochemical effects of titanium dioxide nanoparticles (2 and 5 ppm). Because of this, licorice showed improved resilience to cold stress and significantly lower levels of malondialdehyde and hydrogen peroxide, which resulted in less oxidative damage [128]. The effects of exposure to titanium dioxide on lowering tetracycline (TC) toxicity in rice plants were investigated using rice (*Oryza sativa* L.) and *Arabidopsis thaliana*. Furthermore, because TC sorbs on titanium dioxide, the toxicity of TC in rice roots and shoots was reduced, and this allowed nutrients that were lacking in the presence of TC to be recovered. The phytotoxicity of TC was reduced by the positive and negative effects of titanium dioxide and tetracycline, whereby titanium dioxide nanoparticles abnormally minimised the phytotoxicity [129]. Titanium dioxide nanoparticle application was also investigated for cowpea plants since it was thought to be helpful in raising the amount of chlorophyll in the plants while they were under cadmium stress. Nanoparticles show the ability to reduce plant cadmium levels, which may be the cause of plant stress. Rather, the application of titanium dioxide nanoparticles increased the activity of stress-related enzymes and increased the availability of micronutrients in plants [130]. Additionally, the application of titanium dioxide nanoparticles in tomato plants enhanced the plants’ capacity to withstand stress and produced fruits, biomass and chlorophyll. It also activates enzymes [131] and is used as an alternative for nematicides [132]. As titanium dioxide has a beneficial effect on plant growth as well as human health, it can be used in other areas like medical biotechnology (drug delivery, anti-cancerous activity, anti-microbial activity, phototherapy, bioimaging), agricultural biotechnology (nanopesticide, nanofertilizers, plant stress tolerance) and environmental technology (nanosensors and air/water/soil remediation).

## 5. Nanotoxicity of Titanium Dioxide

**Figure 5:**
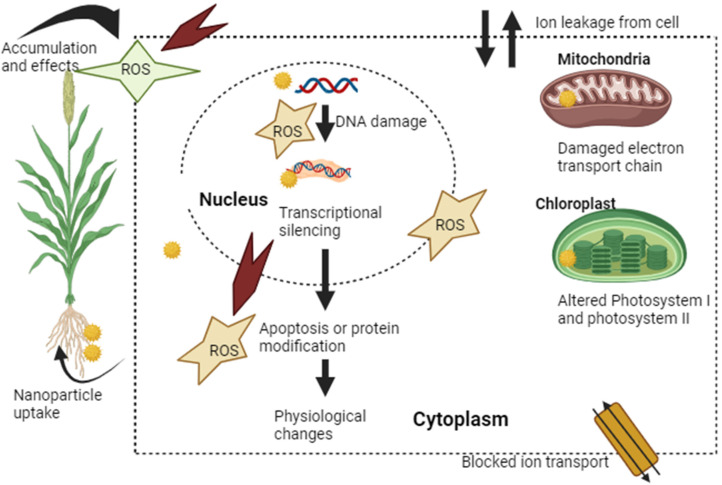
Mechanism of nanotoxicity of titanium dioxide.

Various research has been done that shows the negative and positive effects of titanium dioxide on the growth and development of crops, which refers to the phenomenon of hormesis. This concept of hormesis shows the dual effect based on the concentrations. At low concentrations, nanoparticles show stimulatory effects, whereas at high concentrations, nanoparticles show inhibitory effects [133]. Based on actual evidence and computer models, nanoparticles have been introduced into ecosystems in substantial quantities, which has sparked worries about possible effects on plant development. Titanium dioxide nanoparticles are dispersed into the soil as nano-pesticides and fertilisers, as well as through irrigation and land application of sewage sludge [134]. Because they are producer organisms and are essential to food webs, higher plants are especially important. Numerous studies have looked into the possible harm that titanium dioxide nanoparticles could cause plants (Figure 5).

Numerous parameters, including nanoparticle size, crystal phase, surface coating presence, ambient conditions and plant physiological factors, influence toxicity [121]. According to preliminary research, titanium dioxide nanoparticles may be harmful to plants’ cells and genes, as shown in Table 4 [135]. Titanium dioxide nanoparticles have been shown to have genotoxic effects in *Allium cepa*, where nanoparticles at varying concentrations interact with DNA to harm meristematic cells in roots [136]. When titanium dioxide nanoparticles were applied to onions, malondialdehyde levels rose and root development decreased [137]. A chromosomal abnormality was discovered, which may be related to elevated lipid peroxidation. When titanium dioxide nanoparticles were applied to the roots of *Arabidopsis thaliana*, the microtubular networks were disrupted, causing the root cells to develop isotropically [138]. Titanium dioxide nanoparticles were found to have detrimental effects on *Allium cepa* in a dose-dependent manner by [139]. In *A. cepa* root tips treated with 12.5–100 µg/mL titanium dioxide nanoparticles, the particles enhanced the number of chromosomal aberrations and decreased the mitotic index, as shown in Table 4. Likewise, Fellmann and Eichert [140] found that dose-dependent reductions in titanium dioxide nanoparticle treatment resulted in lower rates of germination and root and shoot growth in maize. According to Kořenková et al. (2017) [141], titanium dioxide nanoparticles have a negative impact on *Hordeum vulgare* root development at increasing concentrations. It was proposed that the hormetic effects of titanium dioxide nanoparticles on *N. arvensis*’s homeostasis would alter the synthesis of proline and the amounts of soluble sugar and chlorophyll [133]. Furthermore, titanium dioxide nanoparticle concentrations were observed to positively correlate with luteolin content structural change; in the presence of 100 ppm titanium dioxide nanoparticles, over 20% of luteolin structure was purportedly affected. Lutein was adsorbed onto the surface of titanium dioxide nanoparticles, as evidenced by the increase in nanoparticle diameter (about 70 nm) and prominent peaks in the Raman spectra [142]. The physicochemical properties of nanomaterials are contingent upon several environmental factors, such as salinity, temperature, light and biological contact. Consequently, they have the potential to gradually alter the environment, which could have a harmful impact on the zoea larvae of *A. lanipes* that vary [143]. Apart from these research studies, TiSiO_4_ nanoparticles significantly increased the levels of progesterone, testosterone, luteinizing hormone (LH), acetylcholine esterase (AChE), lactate dehydrogenase (LDH) activity, lactate dehydrogenase (LDH) activity and follicle-stimulating hormone (FSH) when exposed to rat serum. Conversely, alanine aminotransferase (ALT), aspartate aminotransferase (AST) activity, urea level, immunoglobulins (IgG and IgM) concentrations, progesterone and testosterone levels were significantly decreased. Seven days following exposure, there was a significant increase in the liver comet assay indices. Additionally, the buildup of Silicon and Titanium in the liver, kidney, spleen and lung tissues of the treated rats was noted, as well as histological alterations [144,145].

## 6. Mechanism of Nanotoxicity

Several investigations have been carried out to enhance comprehension of the toxicity mechanism of nanoparticles and their relationship with the surroundings [146]. The reactivity of nanoparticles differs from that of the bulk form, as was previously documented, and one of their primary disadvantages is that we do not fully understand the potential toxicity these particles may generate. One of the most challenging research topics is how nanoparticles might interact and bind to biological systems. Recent research has demonstrated that cells are capable of absorbing nanoparticles with ease; nevertheless, the internal mechanisms involved in this process remain unclear [147]. Nanotoxicology and Ordinary toxicology vary primarily in that the former uses a generic approach with established guidelines for the safer use of nanoparticles, while the latter includes a standardised process for the suitable treatment of nanoparticles [148]. Many in-vitro and in-vivo models, such as *Eudrilus eugeniae*, *Daphnia magna,* etc., have been used to assess the toxicity of nanoparticles such as silver, magnetite, copper oxide, titanium oxide, etc. [149]. Diverse nanoparticles demonstrated distinct harmful attributes and ways of operation. One of the main mechanisms behind nanotoxicity is ROS induction, which results in DNA strand breakage and nucleic acid alteration, oxidative protein modification to create radicals and gene expression modification that reduces a cell’s defence mechanism, genotoxic effect and cell death. There are various mechanisms by which nanoparticles induce reactive oxygen species, and these mechanisms can be surprising at times. Silver nanoparticles were discovered to facilitate ROS generation under various environmental conditions [150]. Titanium dioxide nanoparticles altered the potential of the mitochondrial membrane by photo-catalyzing an early build-up of ROS in cells. Typically, oxidative enzymatic pathways are activated either directly or indirectly by nanoparticles due to their physicochemical reactivity, which generates reactive oxygen species such as hydroxyl radicals and superoxide radical anions [151] primarily the oxidative stress brought on by the subsequent 1. contaminants, such as catalyst-derived transition metals from the production of non-metal nanoparticles, 2. redox-active groups produced when nanoparticles are functionalized; 3. characteristics of the particles alone that produce oxidants. In a different publication, Sahu et al. (2014) [152] stated that they discovered that while HepG_2_ and CaCO_2_ cells were not under any oxidative stress, silver nanoparticles were cytotoxic. Another significant factor that results in toxicity is the cytotoxic effect of nanoparticles, which is contingent upon the quality of the nanoparticles, the mode of delivery and the site of deposition [153]. In reaction to titanium dioxide and silicon dioxide nanoparticles, Sohaebuddin et al. (2010) [154] discovered that three cell lines—RAW 264.7 macrophages, telomerase-immortalized bronchiolar epithelial cells and 3T3 fibroblasts—exhibited a potential cytotoxic mechanism. They deduced that the degree of toxicity and intracellular reactivity are influenced by concentration and size. A 2007 study by Patra et al. [155] found that the physical and chemical properties of the nanoparticles and the kind of cell line influence how hazardous gold nanoparticles are. Cell lines from human liver and lung cancer have also demonstrated differences in toxicity [156]. Zinc oxide nanoparticles have been shown to have potent harmful effects on both mammalian and cells [157]. Shao et al. (2013) [158] report that increased damage and oxidative stress to cell membranes are zinc-based nanoparticles’ most common detrimental impacts on various mammalian cell lines. These findings imply that the nanoparticles might be cytotoxic. According to the studies, cytotoxicity and the production of ROS may be one underlying mechanism underpinning the harmful effects of nanomaterials [151]. Damage to cell membranes results from the interaction of nanomaterials in both situations. Even if the toxicity mechanism is the subject of numerous studies, a deeper comprehension could help reduce the toxic effects of nanoparticles.

## 7. Gene Toxicity of Nanoparticles

Nanoparticles could alter DNA by piercing cell membranes. Gene-toxicity mechanisms can be classified into two basic categories: primary and secondary processes. Single-cell DNA and nanoparticles interact with one another either directly or indirectly in the main process. Direct interactions between a nanoparticle and a chromosome occur during the interphase or mitotic phase. After attaching itself to DNA, the nanoparticle prevents chromosomal breakage and loss (aneugenic effect), transcription and replication. The intermediates of the nanoparticle process release dangerous substances and generate ROS during the indirect interaction. These substances block proteins needed for DNA repair, replication, or transcription. This results in genotoxicity [159,160]. Furthermore, the free radicals cause oxidised base lesions that come from purines and pyrimiMyungdines. These lesions mispair during replication and can lead to dangerous mutations. Furthermore, indirect nanoparticle contact may cause the protein kinases that regulate cell cycle events and division to become inactive, interfering with cell cycle checkpoint functions [161]. The cytotoxicity of zinc, iron and silicon at varying doses was assessed by Cha and Myung [162] using cell lines from the stomach (MKN-1), liver (Huh7), kidney (HEK293), brain (A-172) and lung (A-549). There was a noticeable decline in mitochondrial activity and DNA content in the brain and liver cells. The second mechanism of genotoxicity is the excessive production of ROS by activated phagocytes, such as neutrophils and macrophages, because of a persistent in vivo inflammatory response [160]. This inflammatory reaction affects the surrounding cells by encouraging oxidative stress. These genotoxic pathways have the potential to promote mutagenesis and carcinogenesis as well as chromosomal fragmentation, DNA mutations and changes in the expression of gene profiles [163,164]. Some main elements that affect the genotoxicity produced by nanoparticles include solubility, physicochemical parameters (temperature, pH, etc.), surface coating, particle shape, composition,\ and size [165]. The composition of the nanoparticles is the main element raising the possibility of genotoxicity; for example, the constitution of Cadmium Selenium nanoparticles renders them exceedingly dangerous, irrespective of their size, shape, or route of exposure. Another significant element that influences genotoxicity is particle size. It has a direct impact on the nanoparticles’ reactivity with biological entities, surface-to-volume ratio, solubility and exposure duration. Smaller nanoparticles are more reactive, interfere more with biological systems, generate more ROS and exhibit enhanced genotoxicity as a result [166]. It has been revealed that the primary mechanism of genotoxicity caused by titanium dioxide nanoparticles is oxidative stress. Oxidative stress is a condition characterized by an imbalance favoring the generation of pro-oxidants (ROS) over antioxidant defenses. Excessive formation of reactive oxygen species (ROS), a decrease in GSH and antioxidant levels, or increased lipid peroxidation are all indicators of oxidative stress. Hydrogen peroxide, lipid peroxides, singlet oxygen (1O_2_), hydroxyl and superoxide radicals are among the common compounds forming reactive oxidative species. Although reactive oxidative species are essential for plant and animal health, too much of them can damage cellular components, including proteins, lipids and DNA, endangering the integrity of the cell. In addition to being created by nanoparticles, inflammatory cells such as neutrophils and macrophages can also produce reactive oxidative species through the inflammation-signaling pathway. Lipids may peroxidize because of the reactive oxidative species produced. Lipid peroxidation products have the potential to impact several cellular processes, such as the reduction of cellular GSH. Numerous investigations have linked oxidative stress to nanoparticle-induced genotoxicity. Nevertheless, even though numerous studies have linked the reactive oxidative species that titanium dioxide nanoparticles produce to genotoxicity, DNA damage can be caused by titanium dioxide nanoparticles even in the absence of reactive oxidative species, and DNA damage can also occur when reactive oxidative species is produced by titanium dioxide nanoparticles [167]. There are various research studies that have been done in vivo and in vitro that investigate the genotoxicity of titanium dioxide nanoparticles, but the results are contradictory. Some show positive whereas others show negative results. However, there is no clear explanation for the genotoxicity of titanium dioxide nanoparticles. These inconsistencies in the reports may be due to the use of different concentrations of nanoparticles, exposure time, even due to different sizes, etc. As compared to in vivo, in vitro shows positive results because plants have a defence mechanism, and apart from these mechanisms, there are some ways to reduce the genotoxicity of titanium dioxide nanoparticles, like the green synthesis of nanoparticles, by reducing the exposure time, using coating nanoparticle method, etc. Titanium dioxide nanoparticles can be used as nano-fertilizers because they improve the growth of the plant. The characteristics of the plant rhizosphere, such as the pH of the soil, organic matter, microbes, or rhizosphere exudates, can influence how metal-based nanoparticles behave in their surroundings. Metal-based nanoparticles have the ability to modify the chemistry and microbiological characteristics of rhizosphere soil, hence influencing metal-based nanoparticle behavior and biological impacts. Metal-based nanoparticles, on the other hand, have the potential to harm plant roots or absorb them and create oxidative stress, which can impair the growth and uptake of nutrients by the roots [168].

## 8. Disposal Methods of Nanoparticles

The environment could become contaminated by nanoparticles if they are handled and disposed of improperly since they may have negative effects on biological systems. Additionally, it’s critical to understand the various disposal methods because nanoparticles are being exposed to the environment at an increasing rate. Three strategies can be used to minimize exposure to nanoparticles: personal protective equipment, management strategies and engineering techniques. Most countries have implemented laws that control how nanoparticles are used, handled and disposed of. When managing nanoparticles, researchers have adopted several criteria that must be adhered to. For example, Purdue university’s “Nanoparticle Safety and Health Guidelines” support the use of only normal lab clothing, which includes goggles, lab coats, closed-toe shoes, latex or nitrile gloves and maybe respiratory protection. As to the Swiss government, it is imperative to regulate appropriate safety criteria to enable the prompt and continuous implementation of effective nano-specific protection [169]. The “Guidelines and Best Practices for Safe Handling of Nanomaterials in Research Laboratories and Industries” are equally comprehensive and were released by the Indian government and The Centre for Knowledge Management of Nanoscience and Technology. That study concluded that, in the hazard region designated by the relevant authorities, milligram ranges of nanomaterials should be disposed of in sealed containers that are properly identified and removed using the standard process (Centre for Knowledge Management of Nanoscience and Technology, 2016). The use of precautions when handling nanoparticles, disposing of trash and cleaning processes. Several researchers have proposed several strategies to lower the possibility of environmental contamination caused by NPs. To prolong the life cycle of engineered nanoparticles, for instance, Saravanan et al. (2017) [170] propose that splitting nano waste into two categories—“Intentionally produced engineered nanomaterials (ENMs)” and “Incidentally produced”—is a workable and environmentally responsible approach. Better guidelines for the reuse of nanoparticles can be established as a result. By combining the required ingredients, factory-produced nano waste (produced via a dry process) can be collected, separated and applied externally [170]. In a similar vein, ascorbic acid can be added to cells exposed to nanoparticles to reduce the generation of ROS. Vitamin C, or ascorbic acid, is an antioxidant that can scavenge free radicals. Nanoparticle surfaces can also be modified [171] to reduce nanotoxicity. Currently, several techniques are being investigated to improve the waste management process and stop any possible environmental discharge of nanoparticles.

### 8.1. Recycling of Waste Nanomaterials

Owing to the growing need for tailored nanoparticles, researchers are attempting to recover and reuse nanoparticles in goods. To establish a nanoparticle recycling operation, it is necessary to investigate the recyclability of qualities, including mechanical, chemical and thermal properties, as well as any potential changes in the characteristics of the nanoparticles after recycling. The mechanisms involved in recycling, such as the nanoparticles’ affinity for solid, liquid and gaseous phases; the process’s temperature; and the matrix’s hardness, all affect recycling [172]. Many technological advancements are suggested to enable the recycling of nanoparticles, such as the recovery of catalysts made of gold nanoparticles that can be reused after a reaction cycle [173]. The effects of product contamination or residue from recycled materials on the environment are the primary issues with the recycling process [174]. According to predictions, leftover nanoparticles may be released into the environment and have an impact on the surrounding area [175]. Magnetic ferrite nanoparticles were rendered homogenously dispensable, thermally stable and extremely efficient during the hydroformylation reaction of olefins upon application of an Rh-based cationic complex. This led to a straightforward recovery process. For simple recycling, magnetic nanoparticles were employed as a catalyst [176]. When combined with cloud point extraction, non-ionic surfactants such as Triton X-114 and Triton X-100 made it simple to separate, recover and recycle palladium and gold nanoparticles [177]. Using a microemulsion method, Mdlovu et al. (2018) [178] reclaimed nanoparticles of copper from printed circuit board (PCB) wastes. As these consequences have not been fully investigated, more research is required to understand the implications and impact of recycling nanoparticles better.

### 8.2. Nanomaterial Disposal by Incineration

One extremely promising method of waste management is incineration, which aims to get rid of nanoparticles by altering or managing them through a range of procedures that affect their physio-chemical characteristics or release into the environment [179]. Burning can be used to dispose of pollutants from sewage sludge waste wastewater treatment plants that manage nanoparticles in water, medical wastes, consumer product disposal as municipal solid waste (MSW) and wastes created from nanotechnology research and development [180]. It is imperative to comprehend the behaviour of NPs during the cremation procedure, as their conversion into harmful forms may result in unpredictable consequences. A few investigations have been conducted to learn more about how burning affects nanoparticles. According to a study, depending on the temperature at which they burn and the individual nanoparticles’ melting and boiling temperatures, the designed nanoparticles may be destroyed by full combustion. These temperatures have an impact on how nanoparticles are distributed in the gaseous and solid phases, which determines how much of the material is destroyed [181]. Therefore, it is anticipated that carbon nanotubes can burn entirely while nanoparticles like Zinc oxide, silver oxide and titanium dioxide will reach the gas phase during combustion and turn into bottom ash [182]. The size, oxidation state and chemical composition of the nanoparticles are some of the variables that can impact the success of the incineration process. For instance, depending on their size and aggregation state, reduced particles like aluminium may burn at high temperatures [180]. Nonetheless, some research indicates that some nanoparticles, such as carbon nanotubes and fullerenes, may be able to survive through the combustion zone, which may influence the development of other pollutants. As a result, extra handling and caution are needed to ensure that waste nanoparticle residues do not leak into the environment.

## 9. Conclusions and Future Perspectives

The fields of biotechnology, biomedical sciences, agriculture, medicine and the environment have all seen significant advancements in nanotechnology in recent years. Their contribution to the world’s technological advancements has been significant, and as a result, there is a potential risk of environmental exposure from the expanding usage of nanoparticles in commercial products. Previous research has made it clear that practically all nanoparticles are highly hazardous and have been proven to harm both plants and animals. It has been demonstrated that nanotoxicity can result in cytotoxicity, malignancy, and damage to DNA. In conclusion, titanium dioxide nanoparticles have both harmful and beneficial effects on both plants and animals. In plants, titanium dioxide nanoparticles can be used as nanofertilizers as they improve growth and yield, help plants absorb nutrients and help to cope with stress tolerance. On the other hand, titanium dioxide nanoparticles also induce genotoxicity, which is still contradictory and requires more research. The genotoxicity of the titanium dioxide nanoparticles can be reduced by using different concentrations of nanoparticles, giving less exposure to the plants and the synthesis of nanoparticles from biological extract. Nanoparticles can easily be disposed of by recycling or incineration methods as they are eco-friendly. The focus of study in recent years has been the need for improved evaluation of nanotoxicity and the application of strategies to lower their levels in the environment. The hazards posed by metal nanoparticles have been mitigated with the introduction of biodegradable and biocompatible nanoparticles. The creation of nanoparticles with improved environmental interaction and less harmful effects is currently the main emphasis. From a future perspective, more research should be done to study the role of titanium dioxide nanoparticles in plants. Green synthesis of nanoparticles from the biological extracts should be accepted, as it is cost-effective and less toxic to the environment. The examination of metabolites found in biological extracts should be the subject of more research to ascertain their value for the production of nanoparticles as well as the mechanism of the synthesis of titanium dioxide nanoparticles by green synthesis. More research is required to study the mechanism of toxicity of nanoparticles as different plant species respond to different parameters (size, concentration, exposure time) of titanium dioxide nanoparticles.

## Figures and Tables

**Table 1:** Titanium dioxide nanoparticles synthesis from different plant species.

Plant Species/Parts	Morphological Shape (Size)	Characterization of Titanium Dioxide Nanoparticle [References]
*Aloe vera* (L.)/Leaf	Irregular shape (60 nm)	UV, PSA (Particle size analyzers), XRD, TEM and TGA [40].
*Catharanthus roseus*/Leaf	Cluster form (25 nm)	SEM, XRD and FTIR [41].
*Citrus sinensis*/Peel	Tetragonal shape (19 nm)	PSA, XRD, TEM and TGA [42].
*Cynodon dactylon*/Leaf	Hexagonal shape (13–34 nm)	SEM, XRD and FTIR [43].
*Eclipta prostrata*/Leaf	Spherical shape (36–68 nm)	AFM (Atomic force microscopy), FTIR, FESEM (Field emission scanning electron microscopy) and XRD [44].
*Hibiscus rosa-sinensis*/Flower	Spherical shape	SEM, XRD and FTIR [45].
*Moringa oleifera*/Leaf	Spherical shape (100 nm)	SEM and UV [46].
*Vigna radiata*/Legume	Oval shape	FTIR and SEM [47].
*Piper betle*/Leaf	Spherical (7 nm)	XRD, SEM, UV and FTIR [48].

**Table 2:** Effect of titanium dioxide nanoparticles in various plant species and their gene expression.

Titanium Dioxide Nanoparticles Source/Size/Concentration	Gene Expression	Effect [References]
Chemical/53.18 nm/0.25% Green/64.28 nm/0.1%	Upregulated sodium dismutase (*SOD*) and catalase (*CAT*)	Reduced arsenic toxicity in *Vigna radiata* [77]
Rhawn Company, Shanghai, China/5–10 nm/10 or 20 ppm	Upregulated heat shock protein (HSP17.9 and HSP70) genes	Mitigated the harmful effects of salinity in *Vicia faba* [78]
Nanosany Company, Mashhad, Iran/20 nm/10, 20, 30 or 50 ppm	Upregulated phenylalanine ammonia-lyase (pal) and rosmarinic acid synthase (ras) genes	Enhanced the rosmarinic acid content in *Dracocephalum kotschyi* transformed roots [79]
US Research Nanomaterials, Inc., Houston, TX, USA/5–15 nm/50, 100, or 200 ppm	Upregulated adenylytransferase (*APT*), adenosine-5′-phosphosulfate reductase (*APR*) and sulfite reductase (*SiR*)	Alleviated tetracycline toxicity in *Arabidopsis thaliana* [80]
Macklin Co., Macklin, China/>20 nm/100, 250, 500 or 1000 ppm	U-regulated auxin biosynthesis (*YUC8*), transport (*PIN2*) and signaling (*TIR1*) related genes	Promoted root growth in *Arabidopsis* [81]
Shanghai Chaowei Nanotechnology Co., Ltd., Shanghai, China/70–90 nm/15 ppm	Down-regulated metal transporters, such as OsHMA9, OsNRAMP5 and OsHMA6	Alleviated lead (Pb) toxicity in rice [82]
Sigma-Aldrich, Cat. No. 718467/21 nm/0.1–8 mM	Activated the expression of genes involved in reactive oxidative species (ROS) detoxification/signaling, abscisic acid and salicylic acid signaling pathways	Enhanced resistance to Botrytis cinerea infection, drought and salt stresses in *Arabidopsis* [83]
The source is not mentioned/30–80 nm/5 ppm	Downregulated Cd transporter genes (HMA2 and Nramp5)	Alleviated cadmium toxicity in *Tetrastigma hemsleyanum* [73]
Sigma-Aldrich, Saint-Louis, MO, USA/<100 nm/50 or 100 µg/mL	Upregulated antioxidant enzymes *APX* (Ascorbate peroxidase), *CAT*, *POD* (peroxidases) and *SOD*) gene expressions	Alleviated Polyvinyl chloride (PVC)–microplastics + mercury toxicity in *Pennisetum glaucum* [84]
USA-Nano, Houston, TX, USA/20–30-nm/25 or 50 ppm	Downregulated *GSH1*, *PCS* and *ABC1* genes	Alleviated arsenic toxicity in *Oryza sativa* [85]
Sigma/25 nm/50 or 100 ppm	Upregulated *STR* (Strictosidine synthase), *SGD* (Strictosidine β-D glucosidase), *DAT* (Dopamine transporters) and *PRX* (Peroxiredoxin)	Increased indole alkaloids content in *Catharanthus roseus* [86]
Sigma-Aldrich, USA/>20 nm/100–1000 µg/mL	Upregulated 2-methyl-6-phytylbenzoquinone methyltransferase (*vte5*)	Increased tocochromanol content in *Arabidopsis thaliana* [87]
Sigma Aldrich, St. Louis, MO, USA/21 nm/5, 50 or 150 ppm	Upregulated *SOD*	Reduced shoot growth of *Triticum aestivum* [88]

**Table 3:** Effects of Titanium nanoparticles in the abiotic and biotic stress.

Titanium Nanoparticles Source/Size/Concentration	Effect	Mechanism [References]
Green/30–111 nm/20, 40, 60 or 80 ppm	Mitigated the harmful effects of salinity in *Triticum aestivum*	Not reported [89]
Green/10–100 nm/20, 40, 60 or 80 ppm	Reduced the severity of spot blotch disease in *Triticum aestivum*	Altered agro-morphological characteristics, chlorophyll content, membrane stability, relative water content and non-enzymatic metabolites [90]
Green/<100 nm/20, 40, 60 or 80 ppm	Reduced the severity of yellow stripe rust disease in *Triticum aestivum*	Altered enzymatic and non-enzymatic antioxidants. Upregulated stress-related proteins [91]
Green/30–95 nm/20, 40, 60 or 80 ppm	Under salinity stress, enhanced seed germination, metabolites content and yield in *Triticum aestivum*	Enhanced SOD activity and decreased MDA (Malondialdehyde) content [92]
Green/10–25 nm/30 or 50 ppm	Under salinity stress, enhanced seed germination in *Glycine max*	Decreased Hydrogen peroxide (H_2_O_2_) and MDA content [93]
Green/25–110 nm/25, 50, 75, or 100 µg/ml	Under salinity stress, enhanced seed germination and seedling growth in *Triticum aestivum*	Enhanced activities of POD and SOD and increased free amino acids and proline contents [94]
Green/10–25 nm/50 ppm	Improved tolerance against spot blotch disease in barley	Enhanced chlorophyll content, CAT, POX (Pyruvate oxidase) and Phenylalanine ammonia lyase (PAL) activities and decreased content of H_2_O_2_ and MDA [95]
Green/8–30 nm/15, 30 or 60 ppm Sigma-Aldrich/15 nm/15, 30 or 60 ppm	Green titanium nanoparticles were better than Sigma-Aldrich titanium nanoparticles in mitigating Chromium (VI) toxicity in *Helianthus annuus*	Improved photosynthetic efficiency and antioxidant enzyme activity, decreased oxidative indicators and modified the Ascorbate-Glutathione (AsA-GSH) cycle’s functionality [96]
Iranian Nanomaterial Pioneers Company, Mashhad, Iran/15–20 nm/0.5 or 1.0 mM	Improved ornamental quality of *Catharanthus roseus* under drought	Enhanced carotenoid content, CAT and POD activities and reduced MDA content [97].
Thermo Fisher Scientific/32 nm/100 ppm	Improved drought tolerance in *Lycopersicon esculentum*	Decreased contents of proline and MDA and enhanced photosynthesis-related proteins, plasma membrane intrinsic protein and relative water contents [76]
Degussa GmbH Company, Germany/21 nm/20, 40 or 80 ppm	Improved growth characteristics of *Origanum majorana* under salinity stress	Enhanced free radical scavenging activity [72]
XFNano company, USA/15–25 nm/25, 50, 75 or 100 ppm	Improved salinity tolerance in rice	Increased CAT and POD activities [98]
Aligarh Muslim University, Uttar Pradesh, India/22 nm/50, 100, 150 or 200 ppm	Enhanced growth and essential oil content in *Mentha arvensis*	Enhanced photosynthesis, carbonic anhydrase and nitrate reductase activities [99].
Sigma-Aldrich/21 nm/15 ppm	Improve drought and Ni stress tolerance in *Cucumis sativus*	Enhanced biosynthesis of potassium, hydrogen sulfide and antioxidant (CAT, POX and SOD) enzymes [75]
Sigma-Aldrich, Cat. No. 718467/21 nm/100 ppm	Enhanced UV-B stress tolerance in *Oryza sativa*	Regulates varied biological and metabolic pathways [100]
Sigma-Aldrich, Saint-Louis, MO, USA/<100 nm/40, 80 or 160 ppm	Mitigated the harmful effects of Cd stress and enhanced the yield of *Coriandrum sativum*	Enhanced the content of proline and antioxidative (APX, CAT, Glutathione peroxidase [GPX] and SOD) enzyme activities [74]

**Table 4:** Toxic effect of Titanium dioxide nanoparticles in different plant species.

Plant Species	Particle Size (Concentration)	Toxic Effects
*Lepidium sativum*	Greater than 50 nm (Above 100 mg/kg)	Inhibition of root growth and no effect on seed germination
*Lycopersicum esculentum*	25–29 nm (80 mg/L)	Reduction in the concentration of chlorophyll and increased SOD enzymatic activity
*Oryza sativa*	10–30 nm (2000 mg/L)	Inhibit microbial symbiosis around roots
*Zea mays*	Less than 100 nm and 5 nm (Above 4% in distilled water)	Reduction in root growth and delay seed germination
*Allium cepa*	Less than 100 nm (Above 5 mg/L)	Reduction in chlorophyll synthesis, seed germination and enhanced the chromosomal aberrations.
*Pisum sativum*	15–20 nm, 10 nm (Above 250 mg/L)	Increase in concentration of chlorophyll and enzymatic activity like CAT and APOX in roots and leaves
*Brassica* sp.	Less than 500 nm (Above 1000 mg/L)	Decrease in seed growth and increase in antioxidants

## Data Availability

The data are available within the review.
